# The use of discourse markers in argumentative compositions by Jordanian EFL learners

**DOI:** 10.1057/s41599-023-01525-0

**Published:** 2023-01-30

**Authors:** Anas Huneety, Asim Alkhawaldeh, Bassil Mashaqba, Zainab Zaidan, Abdallah Alshdaifat

**Affiliations:** 1 Department of English Language and Literature, Faculty of Arts, The Hashemite University, Zarqa, Jordan; 2 Department of English Language and Literature, Faculty of Arts and Humanities, Al-alBayt University, Mafraq, Jordan; 3 Mohammed Bin Zayed University for Humanities, Abu Dhabi, United Arab Emirates

**Keywords:** Language and linguistics, Language and linguistics

## Abstract

The aim of the present study is to investigate the use of discourse markers (DMs) in the argumentative compositions written by EFL learners at two academic stages (sophomores and seniors) majoring in English at the Hashemite University, Jordan. The significance of this study springs from its focus on the use of DMs in Jordanian EFL learners’ argumentative writings. Employing an integrated research method of qualitative and quantitative analysis, the findings revealed that both groups of participants used the same types of DMs with varying degree of frequency, namely, elaborative, contrastive, reason, inferential, conclusive, and exemplifier DMs, respectively. The sophomores were observed to employ a relatively higher number of DMs compared to the seniors, which may be ascribed to some redundant instances of DMs. The elaborative, contrastive, and reason types were the most widely used, while inferentials, conclusives and exemplifiers appeared infrequently in both groups. The analysis of individual DMs displayed that the DMs ‘and’, ‘because’, and ‘but’ were the predominant across the seniors and sophomores’ argumentative texts. This overuse of these DMs may be due to the influence of L1 of the participants and the popularity of these DMs among students and teachers of English. Additionally, the participants showed a low proficiency in using DMs since they overused largely a restricted variety of DMs at the expense of others that would be expected in the argumentative writing; some DMs were noticed either to be underused or absent. The results of Pearson’s *r* correlation test indicated that there was a weak positive but significant correlation between the writing quality and the use of DMs. This may be taken as a predictor of the writing quality in argumentative compositions by EFL. Pedagogically, the study emphasizes the significance of teaching DMs, where EFL learners should be taught how to use them appropriately to avoid any transference of their L1. Further research on DMs in argumentative writings in different levels of proficiency is recommended.

## Introduction

Recently, there has been a thriving interest in academic research on linguistic items such as ‘but’, ‘and’, ‘therefore’, ‘because’ (widely referred to as *discourse markers*) that signal the underlying relations that bind units of discourse into a larger cohesive and coherent text (e.g., Aijmer, 2002; Alkhawaldeh, 2018; Andersen, 2000; Beeching, 2016; Blakemore, 2002; Erman, 1987; Fedriani & Sansó, 2017; Foolen, 1996; Fraser, 1999; González, 2004; Heine et al. 2021; Huneety et al., 2017; Jucker, 1997; Lenk, 1998; Lewis, 2003; Olmen et al., 2021; Traugott, 1995). Discourse markers (DMs henceforth) count a functional category that do not typically alter the propositional content of an utterance but play largely an important role in the structuring and organization of discourse. This role that reflects an interpretive relationship between the segment hosting them and the prior utterance can be manifested by means of elaborating or commenting on the prior discourse, indicating a contrast between the foregoing and forthcoming discourse, drawing attention to what is next, reformulating an idea, or highlighting a proposition (Heine et al. 2021).

DMs constitute an indispensably fundamental part of language use, and their pervasiveness in speech and writing makes them a worthwhile object of study. The importance of exploring DMs lies in the fact that they aid discourse cohesion and coherence-they serve as cohesive devices that mark underlying connections between propositions (Al-Khawaldeh, 2018). It has been argued that the use of DMs facilitates the hearer/ reader’s task of interpreting and understanding the speaker/writer’s utterances (Müller, 2005; Aijmer 2015; Schiffrin, 1987; Blakemore, 2002; Huneety, et al., 2019). The adequate use of DMs is pivotal in rendering texts (especially in the context of academic writing) comprehensible and effective. Academic writing that employs DMs is perceived to be more logical, persuasive, and authoritative (Mauranen, 1993). It thus appears that examining DMs in learners’ writing, as the goal of the present study, is a compelling task for the applied linguistics researcher (Siepmann, 2005).

Many studies have highlighted that the use of DMs poses a challenge for EFL learners, especially in writing at colleges and universities. This would be ascribed to a variety of reasons: (i) overuse, underuse, and misuse of DMs are likely to affect the readability and comprehensibility of the text; (ii) the use of DMs is sensitive to text type (e.g., DMs used in argumentative writing differs from those used in expository writing); and (iii) the use of DMs, particularly for EFL learners, tends to vary across languages and cultures (see Altenberg & Tapper, 1998).

The present study investigates the use of DMs in argumentative texts written by two groups of learners at two different levels of proficiency (sophomores and seniors) at the Hashemite University in Jordan. The reason beyond the choice of this type of writing is that it has been characterized as the hardest type in both L1 and L2, in comparison with other types of writings such as narrative and expository (see Yang and Sun 2012).

To achieve the purpose of the present examination, an integrated method of research analysis was employed: quantitative and qualitative. Following Altenberg and Tapper (1998), the comparison in terms of similarities and differences between these two groups of learners was concerned mainly with the overuse and underuse of DMs. These two terms are used in our analysis as purely descriptive labels in the data under examination. Therefore, the misuse of DMs with regard to their incorrect usage (grammar, spelling, punctuation, etc.) is beyond the scope of the present study. Given that, the study seeks to explore the following research questions:

1. Which types of DMs are more or less frequent in argumentative compositions used by EFL learners?

2. Are there any significant differences between sophomores and seniors in the use of DMs in their writing?

3. Is there any correlation between the number of DMs employed in the text and the quality of writing?

The significance of this paper is generally two-fold. First, the insights obtained from the statistical and qualitative findings on how DMs are used by the respective EFL learners would be of some use by teachers and instructors at universities to improve the quality of the learners’ writing performance. Second, the expected findings may offer a better understanding of the correlation between the use of DMs and the quality of writing in Jordanian EFL argumentative composition.

## Review of the literature

This literature review focuses on the general use of discourse markers and the studies conducted on the discourse of EFL learners. Numerous studies have been conducted on DMs and many researchers have investigated the use of DMs by EFL learners in particular e.g., (Martínez, 2004; Jalilifar, 2008; Chapetón Castro, 2009; Aidinlou and Mehr, 2012; Kalajahi, Abdullah, and Baki 2012; Povolná, 2012; Daif-Allah and Albesher, 2013.

Many of these studies compared the use of DMS by EFL learners with that of English learners. These studies have emphasized the poor writing skills of EFL learners, which may be partially attributed to their poor usage of DMs. For example, Altenberg and Tapper (1998) observed that advanced Swedish learners of English underused DMs in their compositions compared to English native students. The most commonly used DMs by Swedish learners were contrastive and inferential ones, while summative DMs (e.g., in sum and short) were rarely used. In a recent study, Tapper (2005) compared the use of DMs by Swedish EFL learners of English to American university students. The findings reported that the Swedish learners of English used far more DMs in their essays than their American counterpart. This overuse of DMs by Swedish leaners of English may be a result of their native language transference which contains more DMs than English does as reported in Altenberg and Tapper (1998). Müller (2005) discussed the use of four DMS (well, you know, like and so) in the speech of German EFL learners’ and native speakers of English. Findings showed that although German speakers used the four discourse markers, some functions were mainly unknown to German speakers who also employed new functions. Fung and Carter (2007) examined the use of discourse markers by Hong Kong learners of English and English speakers. They found that Hong Kong learners widely employed referentially functional DMs (e.g., and, but, because, OK and so), yet they underused a number of DMs such as really, sort of, I see.

Various studies have examined the use and frequency of DMs and their impact of the quality of writing. Some of these studies demonstrated that the frequency of DMs was not an indicator of writing quality. For example, Alattar and Abu-Ayyash (2020) dealt with the use of conjunctions as cohesive devices in Emirati students’ argumentative essays. The study found no positive correlation between the Emirati students’ use of DMs and the quality of their argumentative writing. That is, in many essays, though many participants employed a wide range of DMs correctly, the quality of the texts was poor because it was difficult to understand these texts. Similarly, dealing with the cohesive devices, including connectives in papers written by Chinese learners, Zhang (2021) reported no link between unity of the text and writing quality.

However, some studies reported a correlation between the overuse of DMs. For example, examining the use of DMs in the expository writings by third-year and fourth-year Spanish EFL learners, Martinez (2016) found a positive significant correlation relationship between the density of DMs and the quality of writing. They also revealed that there was little variety in the use of DMs across the both groups of participants.

In the Turkish context, Uzun (2017) examined the use of DMs in argumentative essays written by Turkish EFL learners. In this study, the additive DMs were the most frequent type in the data while adversative and causal types were by far less frequently used. The findings showed a very weak positive relation between the essay scores and writing quality.

Some studies examined the use and frequency of DMs in particular types of texts, showing how each text type prefers some types of DMs. For example, Rahimi (2011) made a comparison between Iranian EFL learners’ argumentative and expository writings. He found that in both types of writing elaborative and contrastive DMs were the most frequently used. In another study, Doró (2016) conducted a study on DMs in argumentative essays written by third-year students of English in the Hungarian university. The study revealed that where the types of DMs with a high percentage of occurrence were elaborative, contrastive and inferential DMs, students tended to underuse summative markers, especially at the end of their essays. Similarly, Ghanbari et al. (2016) drew a comparison between Iranian EFL learners academic and non-academic writings. It was reported that that in academic writings, elaborative and inferential DMs were the predominate, whereas in non-academic writings only elaborative DMs were the most commonly used. In a study on DMs in argumentative and narrative texts written by native and non-native undergraduates, Alghamdi (2014) reported that DMs with a high frequency were elaborative, contrastive, and reason, and there was no significant difference in the use of DMs between both types of writing in the two groups.

An examination of the above literature shows that DMs were investigated in different contexts and in different languages. In the context of Jordan, there have been two studies addressing the use of DMs by Jordanian EFL learners: Ali and Mahadin (2015) and Asassfeh et al. (2013). Ali and Mahadin (2015) studied the use of DMs in expository writing of advanced EFL learners and intermediate EFL learners at University of Jordan. The study has found out that the proficiency level of the student affects the use of DMs. Asassfeh et al. (2013) have investigated the use of logical connectors in expository writings written by Jordanian English-major undergraduates representing the four academic years. They have concluded that students use logical connectors a lot but in an inaccurate way. This study aims to fill in a gap in the literature by examining the use of DMs in a new type of texts, i.e., argumentative compositions, by EFL students. To that end, a sample of 120 students were asked to write compositions that were then analyzed in terms of the use of DMs. What follows is a presentation of the methods employed to collect and analyze data. Results then presented and discussed. The study concludes with some concluding remarks and recommendation for future studies.

## Theoretical Framework

This study draws mainly on Fraser’s (1999) broad characterization of DMs, particularly his taxonomy of DMs. This is because Fraser based his insights on other prominent studies on DMs (e.g., Schiffrin 1987, Blakemore 2002, Redeker (2006)), and his description has been used for written discourse. Fraser (1999) defines DMs as lexical expressions that mostly signal a relationship between S2 and S1, where S2 is the discourse segment which hosts the DM as a part of it, and S1 is the prior discourse segment. Lenk (1998) refers to this function as the prominent textual function of DMs that indicates the kinds of relations existing between different parts of the discourse. DMs come from different grammatical classes, such as conjunctions (e.g., but, also, because,…etc.), adverbs (e.g., furthermore, however,…etc.), prepositional phrases (e.g., on the contrary, on the other hand, as a result …etc. (Fraser, 1999). DMs generally tend to occur in segment-initial position (to introduce an utterance). However, they may also occur medially and finally (ibid).

In this study, six categories of DMs were included for the purpose of analysis. Four of them were adopted from Fraser (1999): contrastive DMs (although, however, yet, etc.) elaborative DMs (and, moreover, in addition, etc.), inferential DMs (therefore, as a result, etc.), and reason DMs (because, since, etc.). The other two categories were suggested by Martínez (2004), namely, conclusives (in conclusion, in short, etc.) and exemplifiers (for example, for instance, etc.).

## Methods and material

The participants of the present study were selected from two different levels of proficiency (sophomores and seniors) at the Hashemite University, Jordan. All of them were EFLs (their native language is Arabic), aging between 18 and 22. During the spring semester 2019–20, a total of 120 students were selected randomly from eight classes.

The students were divided into two groups: the first group consists of 60 sophomores who all passed *basic grammar course* and *paragraph writing course*, and the second group consists of 60 seniors who all passed *advanced grammar course* and *essay writing cours*e. These courses were based on to select the participants of the study, where the former two courses are required for sophomores and the latter two courses are required for seniors. Grammar courses help students gain systemic knowledge of English grammar (the basic and complex grammatical structure of sentences), meanwhile writing courses improve students’ writing ability skills (successful paragraph and essay development)

In order to ensure the homogeneity of the participants in the study and to cover all proficiency levels to get realistic results, the participants were of different gender (male and female) and GPA (ranging from good to excellent). The data of the present study is argumentative texts with a minimum word-count of 250 words on the online-learning. The following task was given to the participants:

‘Online education is rapidly increasing in popularity. Some people think that online teaching is as effective as in-person instruction, while others think online teaching is inferior. Discuss both these views and give your own opinion.’

This topic was chosen in particular because the Hashemite University students have experienced online learning over the past few years. The onset of Covid-19 pandemic has led to such a significant development in online learning. Therefore, we believed that the students are able to argue about this topic easily because they are aware of the advantages and disadvantages of the issue (For details, see also Abdalhadi, et al., 2022).

The researchers used a mixed approach in the present study to address the above-mentioned research questions. After a brief introduction on the importance of online education in Jordan), the students were given 40 min to write the task. All of the 120 paragraphs were examined. The process of identification of DMs in the compiled data draws mainly on Fraser’s (1999, 2006) list of DMs, as presented in Table 1. The reported DMs were calculated in terms of frequency. The wordsmith concordance software (wordsmith tool 4.0) was used for scanning DMs occurrences and generating concordance lists of all DMs detected in the data. For interrater reliability, the compositions were evaluated and scored out of (20 points) by two experienced raters, who are instructors of English. The essays scoring greater than 12 points were assessed as of good quality. The agreement index between the raters reached 95%, which indicates that the scoring was highly consistent among the two raters.

**Table 1:** Categories of DMs.

Category of DMs	Examples
Contrastive	Although, but, however, in contrast, on the other hand, yet.
Elaborative	Also, and, besides, furthermore, in addition.
Inferential	Accordingly, as a result, because of, therefore, thus.
Reason	After all, because, for this/that reason, since.
Conclusive	In conclusion, in short, in sum, to sum up.
Exemplifier	for example, for instance, such as.

To obtain statistical values concerning writing quality, a Pearson’s r correlation test was applied to find out whether the frequency of the use of DMs and writing quality are correlated or not. Pearson’s r correlation is used to measure the strength of a linear correlation between two variables, where the value *r* = 1 means a positive linear correlation, *r* = −1 means a negative linear correlation, and *r* = 0 means no linear correlation. Finally, to compare the results of both groups of students, the means and standard deviations were measured and an independent-samples t-test was carried out to test the significance of difference between the two groups.

## Results and discussion

### Research question 1

#### Which types of DMs are more or less frequent in argumentative compositions used by EFL learners?

The quantitative analysis of data by means of the Wordsmith concordance software showed that the entire data set had a sum of 802 occurrences of 25 different DMs in the argumentative compositions. Tables 2 and 3 show that the participants in the present study used a number of DMs with various rates. All the types of DMs adopted for the present investigation were found to be used by the participants of the present study: elaborative, contrastive, reason, inferential, conclusive, and exemplifier. Although some DMs are by nature poly-functional, particularly ‘and’, all DMs detected in the present data were observed to serve only one function.

**Table 2:** Frequency of DMs classes.

DMs category	Sophomores	Seniors
Elaborative	207 (49.9)	155 (40)
Contrastive	76 (18.3)	104 (26.9)
Reason	68 (16.4)	51 (13.2)
Exemplifiers	27(6.5)	34(8.8)
Conclusive	21(5.1)	31(8)
Inferential	16 (3.8)	12 (3.1)
Total	415(100)	387(100)

**Table 3:** Frequency of individual DMs.

DMs	Sophomores	Seniors
However	11(2.7)	17(4.4)
Although	3(0.7)	6(1.5)
But	40(9.6)	35(9)
Yet	1(0.2)	5(1.3)
In contrast	1(0.2)	14(3.6)
On the other hand	20(4.8)	27(7)
In addition	19(4.7)	20(5.2)
Also	24(5.8)	27(7)
And	150(36.1)	90(23.2)
Besides	6(1.5)	6(1.5)
Furthermore	8(1.9)	12(3.1)
Accordingly	3(0.7)	1(0.3)
As a result	5(1.2)	0(0)
Because of	2(0.5)	8(2.1)
Therefore	3(0.7)	0(0)
Thus	3(0.7)	3(0.8)
After all	0(0)	3(0.8)
Because	59(14.2)	41(10.6)
For this/that reason	9(2.2)	7(1.8)
In conclusion	14(3.4)	22(5.7)
In short	4(1)	2(0.5)
To sum up	3(0.7)	7(1.8)
For example	15(3.6)	10(2.6)
Such as	11(2.7)	24(6.2)
For instance	1(0.2)	0(0)
All	415(100)	387(100)

It can be noticed that both groups used the same types of DMs with varying frequencies. The number of DMs used by both groups revealed that there was no statistical difference, where the total frequency of DMs in the seniors’ argumentative texts was 415 occurrences and 387 in their sophomores’ counterparts. The reason why seniors employed a less number of DMs than did the sophomores may be attributed to the notion that the seniors tended to avoid overusing or redundant instances of DMs observed in the sophomores ‘compositions. This finding is in line with Altenberg & Tapper (1998), who found that the advanced Swedish learners of English used a less number of DMs than non-advanced ones. Moreover, in Yang and Sun (2012) on argumentative essays by Chinese learners of English at two levels of proficiency, sophomores overused DMs more frequently than did seniors. Put it differently, the sophomores slightly outperformed the seniors in terms of the frequency of DMs, which may be due to some redundant instances of DMs, where some DMs were unnecessary and their presence contributed nothing to the text coherence as seen in the examples below. Overall, it can be discerned that both sophomores and seniors had difficulties with using DMs in their argumentative writing in terms of overusing, underusing, omitting, or redundancy. Aijmer (2002) pointed out that learners may underuse or overuse certain forms in their writing in comparison with their native counterparts.

- “Online learning depends on internet and the student himself. And online learning gives us more information and we can search about anything in it, and we can watch the online course more than one time to understand what it is talking about”.
- “Classroom room learning have advantage like you can ask your teacher about anything you can’t understand. and in the same minute, you can share your information and make a conversation about it, and the teacher gives you homework to improve your skill.”

As shown in Table 2, elaborative DMs appeared to be the predominate, compared to other types of DMs, in both groups of learners (sophomores = 49.9% and seniors = 40%). Contrastive DMs ranked the second in the data (sophomores = 18.3% and seniors = 26.9%), followed by reason markers (sophomores = 16.4% and seniors = 13.2%), exemplifiers (sophomores = 6.5% and seniors = 8.8%), and conclusive markers (sophomores = 5.1% and seniors = 8%). The least frequently used type is inferential DMs (sophomores = 3.8% and seniors = 3.1%). It is evident that there was no difference in the rank order of the types of DMs used by both groups of learners.

The statistical findings revealed, as displayed in Table 2, that the three types of DMs (namely elaborative, contrastive, and reason) had a high frequency in the argumentative texts under exploration, in contrast to other types (exemplifier, conclusive, and inferential). Elaborative DMs accounted for the largest percentage of use, followed by contrastive DMs and reason DMs. This is in line with Alghamdi’s findings (2014), which reported that these three categories were utilized at higher rates than other DMs in argumentative compositions in his comparison between NS and NNS students in their narrative and argumentative texts. Likewise, Rahimi (2011) reported in his comparison between argumentative and expository writings by Iranian EFL learners that elaborative and contrastive DMs were used at higher rates than other DMs in argumentative compositions.

This high percentage of the occurrence of elaborative, contrastive, and reason DMs can be attributed to the argumentative mode of the respective texts written by the sophomores and seniors, where they require to employ such DMs for adding new arguments, contrasting ideas, and justifying standpoints. While it was found that the most frequently used types were elaborative DMs, followed by contrastive DMs in the present study, Polish undergraduate learners of English were reported to make more use of the contrastive type than the elaborative one in their argumentative essays (Sanczyk, 2010).

A further analysis of the DMs in the data under examination revealed that elaborative type ranked by far the highest in terms of frequency. It made up 49.9 % of the entire occurrence of DMs in the sophomores’ text and 40% in the seniors’ text. Among this type, the most commonly used DM was, as shown in Table 3, the marker ‘and’, while the other elaborative DMs detected in the data (‘in addition’, ‘also’, ‘besides’, and ‘furthermore’) showed a low frequency, less than 7% for each one in the sophomores and seniors’ data. Table 3 displays that the elaborative DM ‘and’ appeared 150 times with an average of 36.1% by the sophomores, and 90 times with a percentage of 23.2% by the seniors. This is a relatively high percentage that a single item appeared to be taking up almost third of the entire frequency of DMs in the sophomore’s argumentative texts and around quarter in the seniors’ texts.

The overuse of ‘and’ in both groups is not surprising for two reasons. On one hand, preceding research findings report that EFL learners (regardless of their L1) are likely to employ more ‘ands’ than native speakers of English (Taweel, 2020). On the other hand, native speakers of Arabic are inclined to use more ‘ands’ as a result of interference of L1, Arabic, which is characterized with a high frequency of the additive marker *wa:w* ‘and’.

This overreliance on ‘and’ may signal a low proficiency in the use of DMs in the students’ argumentative writing (Uzun, 2017). Strikingly, some elaborative DMs such as ‘moreover’, ‘as well’, ‘too’, ‘or’, ‘in other words’, and ‘further’ were never used in the data. Thus, the overuse of ‘and’ at the expense of other elaborative DMs that were either rarely used or neglected indicates that the learners have a low proficiency level in using DMs.

More elaborately, the overuse of ‘and’ can be due, to a greater extent, to the negative transfer from Arabic as the mother tongue of the leaners, where the DM ‘wa’ ‘and’, in Arabic, is poly-functional that it serves various functions such as, in addition to elaborative, contrastive, causal, and temporal (Hamed, 2014, Arabi & Ali, 2014).

However, it seems that the seniors employed ‘and’ less frequently than the sophomores who largely overused it. This indicates that there was some kind of development/ improvement in the proficiency level of the seniors as they incorporated other elaborative DMs more frequently than those by the sophomores, reducing dependence on ‘and’ in favor of other DMs. Moreover, it is worth mentioning that EFL learners used largely elaborative DMs in argumentative compositions in an attempt to explain, support, and develop their point of view in details, thus, make their thesis statement more well-expressed and persuasive.

Overall measures of DMs in both groups have shown that there is a relative decrease in the occurrences of the elaborative DM ‘and’ by seniors and increase of other elaborative DMs such as ‘also’, ‘furthermore’, ‘in addition’, compared to the sophomores, who used ‘and’ more repeatedly in their texts. That is, there is a common tendency among seniors to employ more frequently diverse elaborative DMs than sophomores.

As for the contrastive DMs that came the second in frequency in both groups, they were frequently used in comparison with other DMs such as, inferential and conclusive categories (the sophomores 18.3% and the seniors 26.9%). By contrast, Jalilfar (2008) reported that the contrastive DMs were the least in the essays written by the intermediate and advanced EFL learners. According to the statistical results, the contrastive ‘but’ was the highest among this class, and it was almost equally used by the sophomores and seniors, ranking third in the total occurrences of the DMs in the data (9.6% and 9%, respectively). This high frequency of ‘but’ may be attributed to the fact that it is very simple in its orthographic structure and semantically unambiguous, which renders it easy for learners to use (Djigunović and Vikov, 2011). Unlike other types of DMs, a variety of contrastive DMs (‘but’, ‘however’, ‘although’, ‘yet’, and ‘on the other hand’) were employed by both groups of learners rather than relying on a very limited number of DMs as the case with the class of reason DMs in this study. Interestingly, the seniors made more use of the contrastive DMs than did the sophomores, which indicates that they have more proficiency and knowledge about the nature of argumentative texts. Given that the argumentation is typically marked by showing a contrast, opposition, and juxtaposition between the argument and counterargument in order to convince the reader/listener of the acceptability of the controversial standpoint at issue (Eemeren, 2021), it was observed that some contrastive DMs that usually appear in argumentative academic compositions such as ‘nevertheless’, ‘nonetheless’, ‘whereas’, ‘conversely’, and ‘despite’ were never used by both groups.

Ranking the third largest category in both groups, the category of reason DMs was relatively moderately used by both groups (16.4% by the sophomores, 13.2% by the seniors). Dissimilar to these findings, it was reported that reason DMs were the most widely used by Turkish learners of English in their argumentative essays (Altunay, 2009). Among this class, the most commonly used one is ‘because’, contrast to other used ones, (after all, and for this/that reason) which were highly underused. Across the both groups, it was the second highest DM, making up 14.2% by sophomores and 10.6% by seniors. One DM, namely, ‘after all’, was used only by the seniors (0.8%), while other DMs, such as ‘since’ was totally absent in both groups. All in all, both groups of learners tend highly to overuse ‘because’ at the expense of other reason DMs, which were largely underused or absent. This could be argued that the learners heavily relied on ‘because’ to compensate for their unfamiliarity with other reason DMs. These results most probably reflect that this type of DMs poses a difficulty for the subjects of the present study.

Less frequently used types in the data were conclusive and exemplifier DMs. The former was used 5.1% by the sophomores and 8% by the seniors whereas, the latter was used 6.5% by the sophomores and 8.8% by the seniors. Only three conclusive DMs (‘in conclusion’, ‘in short’, and ‘to sum up’) were employed by both the sophomores and seniors. Comparing the both groups, the conclusive type had a higher frequency in the seniors’ argumentative texts than the sophomores’ texts. The DM ‘in conclusion’ was the predominate one among this type, while the others were mostly underutilized, where its total frequency in the whole data in both groups was 3.4% by sophomores and 5.7 % by the seniors. Concerning exemplifiers, like the conclusive types, only three DMs (‘for example’, ‘for instance’, and ‘such as’) appeared in the data. The findings showed that the most commonly used one in this class was ‘for example’ in the sophomores’ texts and ‘such as’ in their counterparts. It seems that these two categories appeared more in the seniors’ argumentative compositions. This evidently indicates that there is a development in the seniors’ proficiency level as compared to their counterparts regarding using exemplifiers to give examples as evidence in order to support their argument and conclusives to signal that they have reached the end of the composition and will summarize what has been argued for or against.

As given in Table 2, the least frequently used type by both groups of learners was inferential DMs, accounting for 3.8% by the sophomores and 3.1% by the seniors. This indicates that there was no significant difference between these groups of students with regard to using this type of markers. The analysis showed that the inferential DMs found in the data include ‘thus’, ‘therefore’, ‘because of’, ‘as a result’, and ‘accordingly’. In the inferential category, the DMs ‘as a result’ had the highest frequency in the sophomores’ data, but it was totally neglected in the seniors’ data. While ‘because of’ was the least frequently used by sophomores in the inferential category, it appeared the most frequent one by the seniors. Overall, the students here displayed a tendency to underuse this type of DMs that would be typical in English argumentative writing. This self-evident underuse of inferential DMs in the data under consideration indicates that the learners had insufficient knowledge as such DMs are crucial in texts with argumentative mode. That is, it may be argued that inferential DMs are the most difficult to learn by Jordanian EFL learners.

A closer analysis of the individual DMs used in the argumentative texts written by sophomores and seniors revealed that the most commonly used DMs across both groups of learners were ‘and’ (36.1%) (23.2%), ‘because’ (14.2%) (10.6%), and ‘but’ (9.6%) (9%), respectively. There were no differences in the frequency order of these three DMs between the two groups of leaners. However, they displayed some differences in the number of their occurrence in each group. As shown in Table 3, ‘and’ as an elaborative DM was employed less frequently by the seniors (23.2%) than the sophomores (36.1%). Although this shows that both groups overused this DM at the expense of other DMs, the seniors showed less dependence on this marker in favor of other DMs, which reflects some improvement of their use of DMs, compared to their counterparts. While ‘because’ made a percentage of 14.2% in the sophomores’ writing, it had less percentage in the seniors’ writings (10.6). For the last highest DMs in the data, the contrastive ‘but’, it was equally used by both groups (9.6% by the sophomores and 9% by the seniors). Other DMs occurred by far less frequently such as ‘however’, ‘on the other hand’, and ‘furthermore’. Moreover, there are some DMs that were rarely used by both groups of learners (e.g., ‘although’, ‘yet’, ‘besides’, ‘furthermore’) or were only used by one group (‘as a result’, ‘therefore’, ‘after all’). Remarkably, it was found that an array of manifold DMs was never used neither by the sophomores nor by the seniors (e.g., ‘hence’, ‘nonetheless’, ‘nevertheless’, ‘despite’, ‘on the contrary’, ‘consequently’, ‘since’, ‘in other words’). This can be interpreted that EFL learners tend to rely more on DMs familiar to them from an early stage (Paquot, 2014). Such findings are in agreement with Alghamdi (2014) that in each DM category, there are explicit overuse and underuse of some DMs in EFl argumentative writings.

On the ground of these results, it is justifiable to infer that since both groups of learners in this study utilized frequently a very limited number of DMs in their argumentative texts, they had a poor proficiency level of using DMs, compared to higher proficient L1/ L2 writers (Zhang, 2021). The importance of such findings stems from the fact that the quality of academic writing can be evaluated based on lexical variety (Hinkel, 2004).

### Research question 2

#### Are there any significant differences between sophomores and seniors in the use of DMs in their writing?

To address the second question of the present study, an independent-samples *t*-test was undertaken in order find whether there is any statistically significant difference between the sophomores and the seniors’ use of DMs in their argumentative written texts. As displayed in Table 4, the results of the respective test revealed no statistically significant difference between the sophomores (*M* = 69.17, *SD* = 72.07) and seniors (*M* = 64.50, *SD* = 54.29) in their use of DMs in argumentative papers; *t*(10) = 0.126, *p* = 0.901.

**Table 4:** The results of use of DMs in argumentative compositions.

	M	SS	SD	Df	N
Sophomores	69.17	25970.83	72.07	5	6
Seniors	64.50	14741.50	54.29	5	6

Abbreviations: *M* mean, *SS* sum of squares, *SD* standard deviation, *Df* degrees of freedom, *N* sample size. Note: *Significant difference at *p* < 0.05.

More elaborately, the total occurrences of the DMs found across argumentative compositions written by the two groups in this study was 802, as clearly illustrated in Table 5 below, where they had a frequency of 415 occurrences in the sophomores’ texts and 387 occurrences in the seniors’ texts. It can be seen that the frequency of DMs in the sophomores’ compositions was slightly higher than the seniors’. The present study conducted a lexical density test (a test used to measure the proportion of the content (lexical) words over the total words) to measure the proportion of the DMs to the total number of words (the total number of words in the sophomores and seniors’ data is 8549 and 8991, respectively) in the argumentative data under examination. The numerical results displayed that the lexical density (LD), which refers to the proportion of DMs to the total number of words, is 4.8% in the sophomores’ writings and 4.3% in the seniors’ writings. It has been reported that the density of DMs and quality of writing are positively related in EFL learners’ compositions (Martínez, 2016). In this regard, the current results can, to some degree, may indicate that the participants showed a low proficiency in writing their argumentative texts. A number of studies report that more proficient learners tend to use an increased amount of DMs in their written texts (see Uzun, 2017).

**Table 5:** Frequency of DM occurrences in argumentative compositions.

Grade	Frequency and (percentage)
Sophomores	415(51.7)
Seniors	387(48.3)
All	802(100)

### Research question 3

#### Is there any correlation between the number of DMs employed in the text and the quality of writing?

With regard to the last question of the study concerning the relationship between the frequency of DMs and the quality of writing, a Pearson’s *r* correlation test was carried out to assess this relationship. As illustrated in Table 6, the results displayed that the correlation between the frequency of DMs employed in the argumentative compositions written by the sophomores and their evaluation was weakly positively correlated, *r*(58) = .32, *p* = 0.012. Likewise, the frequency of DMs and the evaluation of the seniors’ writings were found to be weakly positively correlated, *r*(58) = 0.42, *p* < 0.001. Based on the results obtained from the present correlation test, it can be stated that a positive correlation but significant (the sophomores 0.012656 and the seniors .000764) was found between the total use of DMs and the quality writing of the argumentative texts written by the participants of the present study. It can be suggested that the highly-rated argumentative compositions tend to employ more DMs than did their poorly-rated counterparts.

**Table 6:** The results of Pearson’s *r* correlation test.

		Sophomores	Seniors
		DMs Freq.	
Evaluation	Pearson correlation	0.3201	0.4228
	Sig.	.012656*	.000764**
	N	60	60

Abbreviations: *Freq.* frequency, *Sig.* significance, *N* the number of data points. Note: *The result is significant at *p* < 0.05. **The result is significant at *p* < 0.01.

Although the results of the present study revealed that there was a positive correlation between the two values at issue, the relationship between the frequency of DMs and the evaluation was weak (for the nearer the value is to zero, the weaker the relationship). However, we can infer that the frequency of DMs can be, to some extent, a potential predictor/ indicator of writing quality, that is, the higher the number of DMs, the better the quality of writing. Such findings are in line with the studies that support the existence of a positive correlation between the deployment of DMs and the quality of writing (Jin, 2001; Liu and Braine 2005, Yang and Sun 2012). This implies that EFL learners of both groups in this study still face some difficulties in using DMs in their argumentative writing. The absence of significantly positive correlations between the quality of writing and the frequency of DMs in the respective argumentative texts reflects the students’ low-level proficiency in employing DMs.

However, it should be borne in mind that correlational tests do not always suggest causation- that when two variables in tandem do not necessarily indicate that one variable is affecting the other. (Bruce &. Harper, 2012).

## Conclusion

The present study examined the use of DMs in argumentative writing by the seniors and sophomores majoring in English at the Hashemite university. These two groups of EFL learners represented two different level of proficiency. The findings revealed both groups used the same types of DMs with varying degree of frequency: elaborative, contrastive, reason, inferential, conclusive, and exemplifier. The seniors were found to employ more slightly DMs than did the sophomores, which may be a result of overusing some DMs and unnecessary instances of DMs. There was no statistically difference in the frequency of DMs by both groups.

The types of DMs that appeared commonly were elaborative, contrastive and reason. However, conclusives and exemplifiers were infrequently used. Across the both group of data, the elaborative type of DMs was the predominate. The analysis of individual DMs reported that the DMs ‘and’, ‘because’, and ‘but’ were the most widely used in both groups. It also reported that both groups over-relied on a very limited number of DMs in their argumentative writing at the expense of other DMs, which reflects a low proficiency in using DMs.

Moreover, there are some DMs that were rarely used by both groups of learners (e.g., although, yet, besides, furthermore) or were only used by one group (as a result, therefore, after all). Remarkably, it was found that an array of manifold DMs was never used neither by the sophomores nor by the seniors (e.g., hence, nonetheless, nevertheless, despite, on the contrary, consequently, since, in other words).

The findings indicated that there was a weak positive but significant correlation between the use of DMs and the quality of writing in both argumentative texts written by the sophomores and seniors.

### Pedagogical implications

Based on the present findings on the use of DMs in the sophomore and seniors’ argumentative writings, some pedagogical implications can be highlighted. As we have seen, the use of DMs in argumentative writings presents a challenge to EFL learners across different levels of proficiency. Moreover, the analysis reveals that EFL learners demonstrate little variety in the use of DMs.

The inappropriate use of DMs should be attended to by both instructors and learners. More focus should be placed on DMs and students should be exposed to more varied DMs. In other words, instructors of English should familiarize their students with a wide variety of DMs and encourage learners to vary in their choice of DMs in their writings rather than relying on restricted range of DMs. To increase the quality of EFL argumentative writing, learners should be given more exercises on the functions of DMs and their role in creating and maintaining the cohesion and coherence of text, especially, in academic writing (For details, see Guba et al., 2021). This would help in the development of the EFL learners’ writing proficiency.

## Data Availability

The datasets generated during and/or analyzed during the current study are available from the corresponding author on reasonable request.
